# Maternal Kisspeptin-10 Treatment Partially Rescues Fetal and Postnatal Cardiac Programming Disrupted by Maternal Hypothyroidism

**DOI:** 10.1007/s12012-026-10185-w

**Published:** 2026-09-11

**Authors:** Erikles Macedo Barbosa, Natalia Panhoca Rodrigues, Bianca Reis Santos, Jeane Martinha dos Anjos Cordeiro, Thayna Queiroz Menezes da Silva, Cibele Luz Oliveira, Luciano Cardoso Santos, Maria Clara da Silva Galrão Cunha, Rogeria Serakides, Juneo Freitas Silva

**Affiliations:** 1 Centro de Microscopia Eletrônica, Departamento de Ciências Biológicas, Universidade Estadual de Santa Cruz, Campus Soane Nazaré de Andrade, Ilhéus, 45662-900 Brasil; 2 Departamento de Clínica e Cirurgia Veterinária, Escola de Veterinária, Universidade Federal de Minas Gerais, Av. Antônio Carlos, 6627, Belo Horizonte, 31270-901 Minas Gerais Brasil

**Keywords:** Kiss1, Heart, Fetus, DOHaD, Thyroid, PTU

## Abstract

Cardiovascular diseases are the leading cause of global mortality and have been associated with alterations in fetal programming. Maternal hypothyroidism (MH) impairs placental function and induces intrauterine growth restriction (IUGR), both of which are risk factors for cardiovascular disease. Kisspeptin-10 (Kp10) has been shown to improve feto-placental development in hypothyroid rats, but its effects on intrauterine cardiac development remain unknown. MH was induced in Wistar rats using propylthiouracil (PTU), and Kp10 administration began on gestational day 8. Offspring hearts were analyzed at fetal day 18 and postnatal days 3 and 21. MH reduced fetal and postnatal body and heart mass, impaired cardiomyocyte proliferation, and dysregulated apoptotic and angiogenic markers. Maternal Kp10 administration enhanced postnatal weight gain, restored cardiomyocyte proliferation and nuclear density, and positively modulated apoptotic (*Bax*/*Bcl2*) and angiogenic (Vegf, Ang2, Flk1) pathways. However, it increased redox (8-OHdG) and endoplasmic reticulum (ER) stress (*Grp78*, Chop) mediators in fetal hearts, while reduced postnatal Chop expression in both sexes. Collectively, these findings demonstrate that maternal hypothyroidism disrupts cardiac development and postnatal cardiac programming, whereas maternal Kp10 administration partially mitigates these effects, highlighting novel mechanisms through which kisspeptin may positively regulate cardiac development.

**Graphical abstract:**

**Figure:**
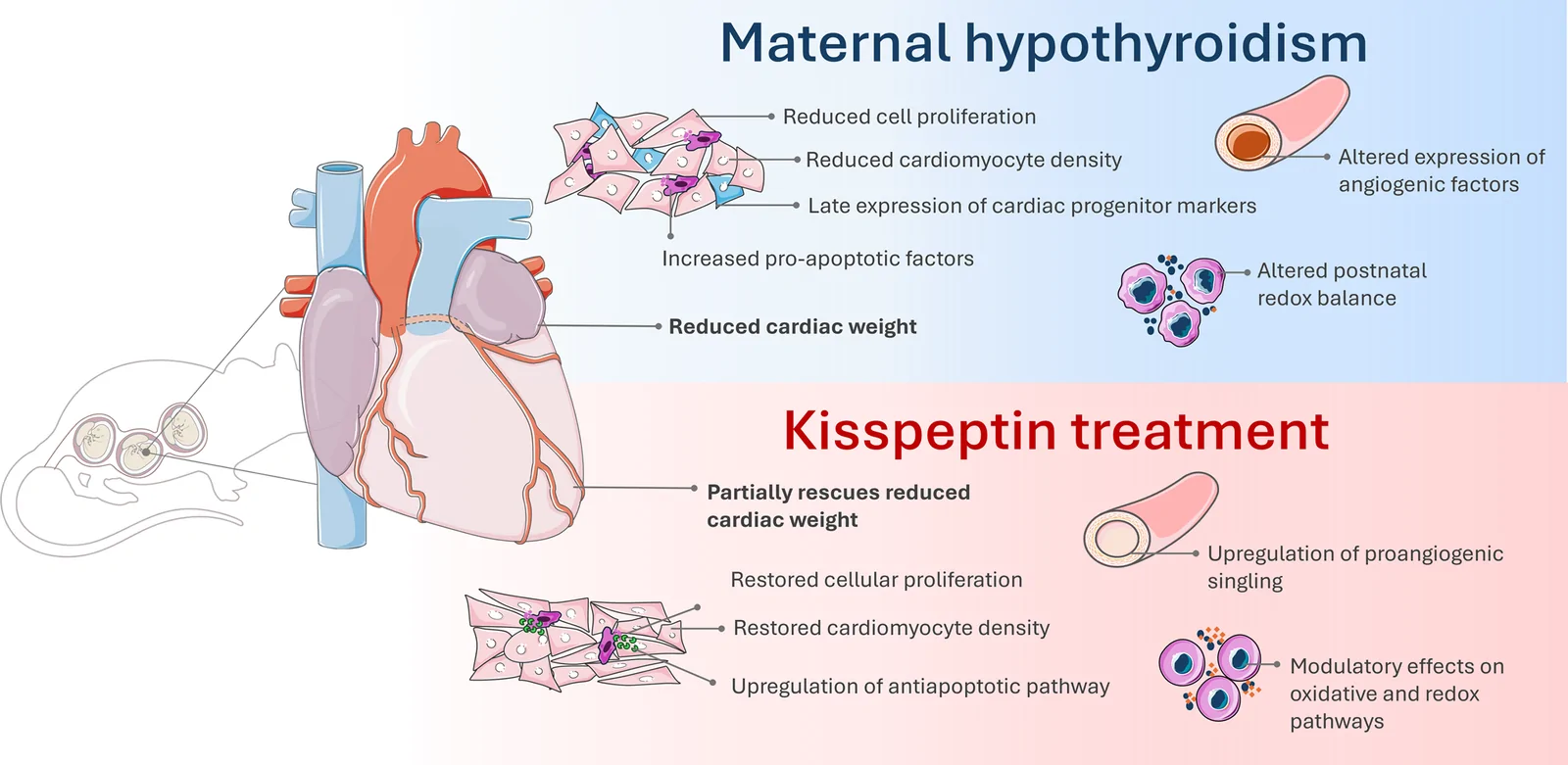

**Supplementary Information:**

The online version contains supplementary material available at https://doi.org/10.1007/s12012-026-10185-w.

## Introduction

Cardiovascular diseases (CVDs) remain the leading cause of worldwide mortality, posing a persistent challenge for health systems [1]. Although multiple etiologies have been proposed for the development of CVDs, it is well established that fetal programming of cardiac development is a central factor in the origin of heart disease [2]. Barker’s pioneering studies showed that adverse maternal conditions can alter the intrauterine environment, leading to impaired fetal programming, intrauterine growth restriction (IUGR), and increased susceptibility to cardiovascular disease in adulthood [3, 4].

Maternal hypothyroidism (MH) is one of the main endocrine disorders of pregnancy. Women with this condition have a higher incidence of recurrent miscarriage and prenatal complications such as pre‑eclampsia, preterm birth and, particularly, IUGR [5, 6]. In animal models, MH compromises placental morphophysiology, impairing maternal–fetal communication, and placental insufficiency is the primary cause of IUGR [5, 7–10]. However, despite the known MH–IUGR relationship, the molecular mechanisms underlying alterations in fetal organogenesis and postnatal outcomes, especially cardiac development and lifelong cardiovascular disease programming, remain poorly explored.

The search for effective therapeutic targets to prevent cardiovascular disease is ongoing; however, few studies have considered fetal programming as a central target of these interventions. In this context, reduced kisspeptin concentrations in the plasma of women have been observed in IUGR, suggesting a possible role for this peptide in normal fetal development [11, 12]. We recently demonstrated that MH in rats is associated with reduced placental kisspeptin signaling on the placenta [13] and that its administration during gestation improves placental morphology and function, positively impacting fetal development [14]. Although kisspeptin is well known for regulating the reproductive axis [15] and has been proposed as a therapeutic target for various reproductive dysfunctions in both humans and animals, it is also expressed in the heart [16], but its role in this organ remains poorly understood.

Studies have shown that administering kisspeptin to female rats alters cardiac structure, increasing collagen deposition and changing mitochondrial morphology, and modifies serum metabolite levels and global cardiac gene expression [17]. Reduced myocardial kisspeptin levels have also been reported in human patients with ischemic heart disease [16]. Despite this evidence, the role of kisspeptin in fetal and postnatal cardiac programming remains unexplored. We hypothesized that the reduction in placental kisspeptin levels observed in IUGR pregnancies, as in MH [13], is associated with alterations in fetal cardiac development, with changes in the pre‑ and postnatal redox and angiogenic state, and that the administration of kisspeptin during gestation can attenuate these effects. Thus, this study aimed to evaluate morphology, proliferation, and the expression of apoptotic, redox, and angiogenic mediators in the fetal heart and in the hearts of offspring of hypothyroid rats treated with kisspeptin during pregnancy. This is the first study to characterize MH’s negative effects on the proliferative, apoptotic, and angiogenic status of the fetal and offspring heart, showing sex‑ and age‑dependent patterns, and to demonstrate the cardioprotective role of kisspeptin in fetal programming under conditions of thyroid complications.

## Materials and Methods

### Animals

Adult Wistar rats (*n* = 48; 204 ± 14 g) from the Animal Breeding, Maintenance and Experimentation Laboratory (LaBIO) at the State University of Santa Cruz (UESC) were housed in plastic cages (6 animals/cage) under a 12 h light/12 h dark cycle at 22 ± 2 °C. Food and water were provided *ad libitum*. All procedures were approved by the UESC Ethics Committee on the Use of Animals (CEUA/UESC; Protocol No. 27/22) and were performed according to the ARRIVE guidelines (Animal Research: Reporting of In Vivo Experiments - https://arriveguidelines.org/) and the International Council for Laboratory Animal Science (ICLAS).

### Experimental Design

After 7 days of acclimation and/or confirmation of two consecutive estrous cycles, female rats received 4 mg/kg/day of 6‑propyl‑2‑thiouracil (PTU) by orogastric gavage (Sigma‑Aldrich, St. Louis, MO, USA) to induce hypothyroidism, while control animals received water as placebo [13, 18]. From the 4th day of administration, rats in proestrus were housed overnight with healthy males, and the presence of spermatozoa in the next morning’s vaginal cytology confirmed mating, designated gestational day 0 (GD0). PTU or water treatment continued until the end of gestation.

On GD8, half of the hypothyroid rats began receiving daily intraperitoneal Kisspeptin‑10 (Kp10; 8 µg/kg; Tocris Bioscience, Bristol, UK), while the others received sterile water as placebo [13, 18]. Thus, three groups were formed: Control (*n* = 16), Hypothyroidism (*n* = 16) and Hypothyroidism + Kp10 (*n* = 16). Eight rats from each group were euthanized on GD18 by decapitation, and blood was collected for free T4 analysis. The animals were laparotomized, and the utero‑placental units were isolated and dissected. The number of viable fetuses, as well as fetal mass and length, were assessed. The fetuses were then decapitated, and two fetuses per dam were randomly selected and dissected to collect the entire heart. Samples were immersed in Trizol (Invitrogen, Life Technologies, Carlsbad, CA, USA) and stored at − 80 °C for subsequent RT‑qPCR. The remaining fetuses were dissected to collect and weigh the hearts. Three hearts per litter were randomly selected, fixed in 4% paraformaldehyde (PFA) at 4 °C for 24 h, and processed by paraffin embedding for histological and immunohistochemical analyses.

The remaining eight rats in each group gave birth around GD21. At this point, postnatal day 0 (PND0) was established. All pups were weighed every three days until PND21 to obtain the body mass gain curve. On PND3, litters were randomly culled to eight pups per dam (4 males and 4 females), and surplus pups were euthanized by decapitation. Blood from the euthanized pups (pooled per litter) was collected in a heparinized microtube for free T4 analysis. These pups were then dissected to collect and weigh their hearts. Samples of the heart apex—including the right and left ventricles—from one male and one female were collected and stored in Trizol at − 80 °C for RT‑qPCR. A second sample of these tissues was fixed in 4% PFA at 4 °C for 24 h and subsequently paraffin‑embedded for histological and immunohistochemical analysis.

On PND21, the dams and litter were euthanized by decapitation. Blood samples were collected for free T4 analysis, and pups were dissected to collect and weigh their hearts. Samples from one male and one female per litter were randomly selected and collected as described for PND3 for gene, histological and immunohistochemical analyses.

### Hormone Dosage

Blood from dams and pups (PND3 and PND21) was collected into heparinized tubes and centrifuged at 3,000 rpm for 20 min at 4 °C to obtain plasma, which was stored at − 20 °C until hormonal assays. Free T4 was quantified by ELISA (sensitivity: 0.4 ng/dL) using commercial kits, according to the manufacturer’s instructions (IMMULITE, Siemens Medical Solutions Diagnostics, Malvern, PA, USA). The intra‑ and inter‑assay coefficients of variation were 4% and 7%, respectively.

### Immunohistochemistry

Heart samples were paraffin‑embedded and 4 μm sections mounted on silanized slides (StarFrost Polycat, Germany) were processed using the streptavidin–biotin–peroxidase method with the NovoLink Kit (NOVOLINK POLYMER‑DETECTION‑SYSTEMS, Leica Biosystems Inc., Buffalo Grove, IL, USA) [18]. Antigen retrieval was performed in a 98 °C water bath with slides immersed in citric acid buffer (pH 6.0) for 20 min. Endogenous peroxidase was blocked with 3% hydrogen peroxide in methanol. Nonspecific binding sites were blocked with the kit’s blocking solution. Slides were incubated in a humid chamber for 18 h (overnight) with the primary antibody (Supplemental Table 1). Slides were then incubated with a secondary antibody (rabbit anti‑mouse IgG) for 45 min in a humid chamber, followed by streptavidin–peroxidase for 30 min. Between each step, slides were washed three times with phosphate‑buffered saline (PBS; pH 7.2). The chromogen used was diaminobenzidine (EnVision FLEX DAB+ Chromogen; Agilent Technologies, Inc., Santa Clara, CA, USA). Incubation times were adjusted for each primary antibody (Supplemental Table 1). Sections were counterstained with Harris hematoxylin. Negative controls were obtained by replacing the primary antibody with PBS. Positive controls included rat placenta and decidua at GD14 for Vegf, Flk1, 8‑OHdG, Sod1, Cat, Gpx1, Chop, Kiss1 and Kiss1r, and fish ovary for Ang2 (Supplemental Figs. 6 and 7) [13, 18, 19].

Immunohistochemical expression was evaluated both descriptively and quantitatively in five randomly selected images of the left ventricular wall acquired at 40× magnification using a Leica DM2500 light microscope (Leica Microsystems, Wetzlar, Germany). The immunopositive area was quantified using ImageJ® software (U.S. National Institutes of Health, Bethesda, MD, USA) by colour deconvolution followed by image thresholding, according to a previously described automated analysis macro ⁷¹. The data were recorded, analysed, and expressed as the immunostained area in pixels ⁷¹.

### RNA Extraction and RT‑qPCR

RNA was extracted from the samples using Trizol, and RNA concentration and quality were assessed with a NanoDrop 2000 spectrophotometer (Thermo Scientific). For RT‑qPCR, cDNA was synthesized from 1 µg of RNA. Reverse transcription reactions used the GoTaq® qPCR and RT‑qPCR Systems Kit (A6010, Promega). Transcripts of target genes for transcription factors (*Isl1*, *Fgf8*, *Foxh1*), apoptosis (*Bax*, *Bcl2*), the Kiss system (*Kiss1*, *Kiss1r*), angiogenesis (*Vegf*, *Ang2*, *Flk1*) and redox status (*Sod1*, *Cat*, *Gpx1*, *Grp78*, *Chop*) were quantified by qPCR using SYBR Green on an Applied Biosystems® 7500 Real‑Time PCR System. The primers used are listed in Supplemental Table 2. Each reaction contained 1.5 µL cDNA, 100 nM of each primer and 12.5 µL GoTaq® qPCR Master Mix (2X) in a final volume of 20 µL. Negative controls were prepared by replacing the cDNA sample with DNase‑/RNase‑free water. Cycling conditions were: enzyme activation at 95 °C for 2 min; 40 cycles of denaturation at 95 °C for 15 s and annealing/extension at 60 °C for 60 s. qPCR linearity and efficiency were evaluated using standard curves generated from serial cDNA dilutions, and melting curves were analyzed to confirm product specificity. Gene expression was calculated by the 2^−ΔΔCT^ method. Results for each group were quantified after normalization to *Gapdh* (glyceraldehyde‑3‑phosphate dehydrogenase) expression in *Rattus norvegicus*, using the control group as reference [18]. For the *Foxh1* gene, which was not expressed in the control group, the hypo group was used as reference.

### Statistical Analysis

All data were first tested for normality (Shapiro–Wilk) and homoscedasticity (Brown–Forsythe). Data that did not meet assumptions were log‑transformed for parametric analyses. All analyses were performed by ANOVA followed by the Student–Newman–Keuls (SNK) test. Analyses were carried out in GraphPad Prism 8.0.2, and differences were considered significant at *P* < 0.05.

## Results

### Maternal Kp10 Administration Increases Body Mass Gain in the Offspring of Hypothyroid Rats

**Fig. 1:**
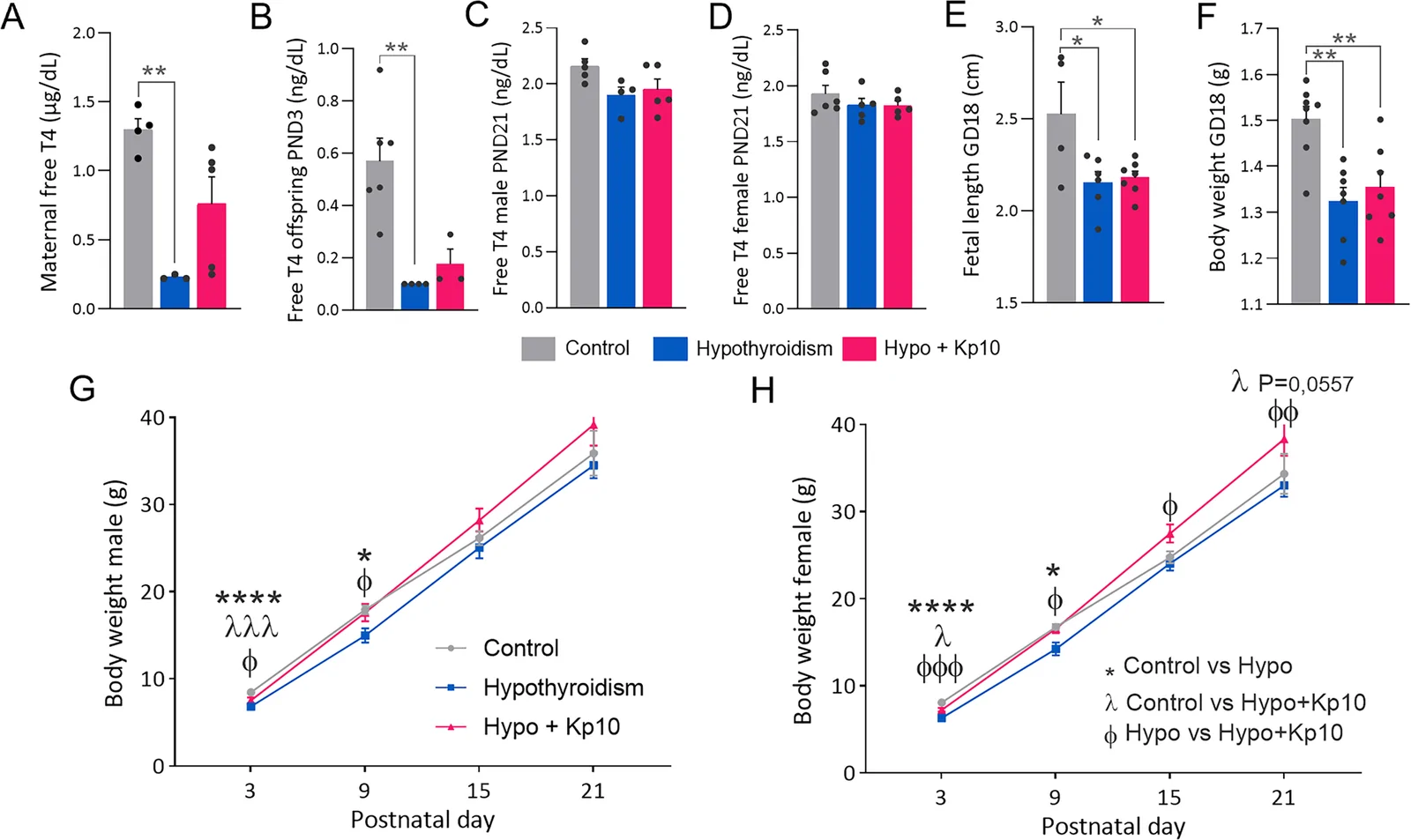
Confirmation of hypothyroidism induction and monitoring of body mass gain in offspring from the Control, Hypothyroidism and Hypothyroidism + Kp10 groups. **A** Maternal free T4 concentration (µg/dL) at GD0 (*n* = 3–5/group). **B** Free T4 concentration (ng/dL) in PND3 offspring (pooled, *n* = 3–6/group). **C** Free T4 concentration (ng/dL) male PND21 (*n* = 5–6/group). **D** Free T4 concentration (ng/dL) female PND21 (*n* = 5–6/group). **E** Fetal length (cm) (values represent the mean per litter [*n* = 4–7 dams/group]) (mean ± SEM). **F** Body mass (g) of fetuses on GD18 (values represent the mean per litter [*n* = 7 dams/group]). **G** Growth curve of male offspring (*n* = 7/group). **H** Growth curve of female offspring (*n* = 7/group). (Mean ± SEM). Significant differences are indicated by *P < 0.05, **P < 0.01, ***P < 0.001, and ****P < 0.0001 for one-way ANOVA followed by SNK post hoc test

Hypothyroidism induction was confirmed by reduced plasma free T4 levels in PTU‑treated rats compared with controls (Fig. 1A; **P < 0.01). Offspring of hypothyroid rats also showed lower free T4 at PND3 versus controls (Fig. 1B; **P < 0.01). Maternal Kp10 administration did not statistically change free T4 concentrations in dams or offspring (*P* > 0.05), despite the visually higher levels observed in this group. Fetuses from hypothyroid rats were also smaller and had lower body mass on GD18 compared with controls (Fig. 1E, F; **P < 0.01, ***P < 0.001). Kp10 administration did not reverse the MH‑induced reduction in fetal length and body mass (Fig. 1E, F; *P < 0.05, **P < 0.01).

Offspring growth was monitored throughout lactation. At PND3 and PND9, body mass was lower in hypothyroid group than controls regardless of sex (Fig. 1G, H; *P < 0.05; **P < 0.01; ****P < 0.0001), while maternal Kp10 increased body mass versus the hypothyroid group at PND9 (Fig. 1G, H; *P < 0.05; ***P < 0.001), matching control values (*P* > 0.05). From PND15 onward, no significant differences were seen in male body mass between groups, whereas female offspring from the Kp10-treated group weighed more than those from the hypothyroid group at PND15 and PND21 (Fig. 1G, H; *P < 0.05; **P < 0.01). Females in the Kp10 group showed a trend toward higher body weight than controls at PND21 (Fig. 1H; *P* = 0.0557).

### Kp10 Administration Restores the Reduced Proliferative Activity in Fetal Cardiomyocytes Caused by Maternal Hypothyroidism

**Fig. 2:**
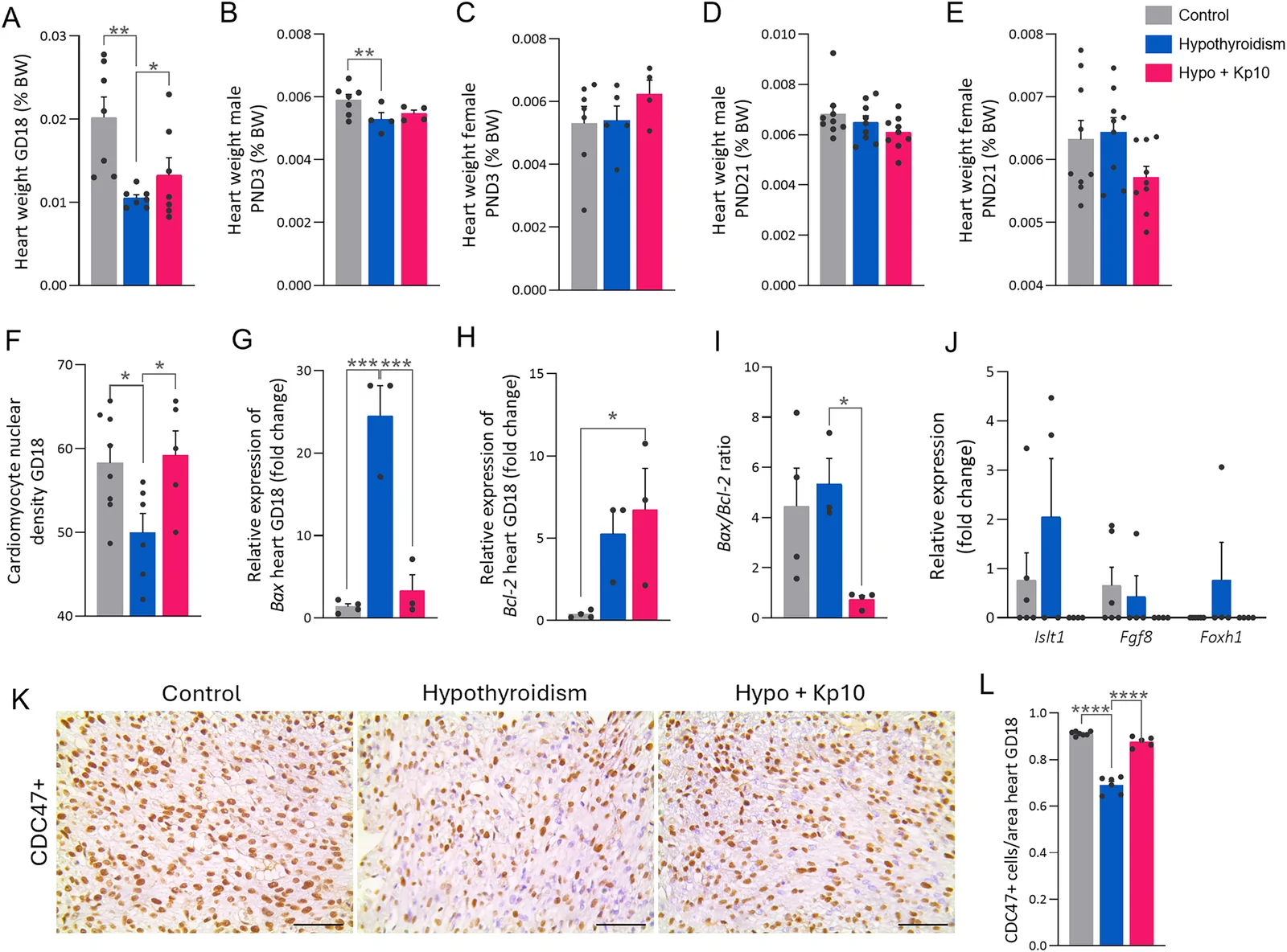
Cardiac development of the offspring and cell proliferation in the fetal heart of Control, Hypothyroidism and Hypothyroidism + Kp10 groups. **A** Cardiac mass of GD18 fetuses (% body mass, values represent the mean per litter [*n* = 7 dams/group]). **B** Cardiac mass of male PND3 (% body mass, *n* = 7/group). **C** Cardiac mass of female PND3 (% body mass, *n* = 7/group). **D** Cardiac mass of male PND21 (% body mass, *n* = 9/group). **E** Cardiac mass of female PND21 (% body mass, *n* = 9/group). **F** Cardiomyocyte nuclear density at GD18 (number of cardiomyocyte nuclei/area, *n* = 5–7/group). **G** Relative *Bax* gene expression in the GD18 fetal heart (*n* = 3–4/group). **H** Relative *Bcl‑2* gene expression in the GD18 fetal heart (*n* = 3–4/group). **I** *Bax/Bcl‑2* ratio in the GD18 fetal heart (mean ± SEM). **J** Relative *Isl1*, *Fgf8* and *Foxh1* gene expression in the GD18 fetal heart (*n* = 4–6/group). **K** Photomicrographs of CDC47 immunohistochemical staining in the GD18 fetal heart (streptavidin–biotin–peroxidase; Harris hematoxylin; Bar= 50 μm). **L** Number of CDC47 + cells/area in the GD18 fetal heart (×10^5^) (*n* = 5–7/group) (Mean ± SEM). Significant differences are indicated by *P < 0.05, **P < 0.01, ***P < 0.001, and ****P < 0.0001 for one-way ANOVA followed by SNK post hoc test

Fetal growth restriction is linked to cardiac disease programming [3, 4, 20, 21], and the number of fetal cardiomyocytes is proportional to the number of cardiac cells in the adult heart [22]. In our model, MH reduced fetal heart mass (**P < 0.001) and cardiomyocyte nuclear density (*P < 0.05) compared with controls (Fig. 2A, F). Maternal Kp10 administration did not alter heart mass but restored cardiomyocyte nuclear density to control levels (Fig. 2F; *P < 0.05). Male offspring in the hypothyroid group still had lower heart mass at PND3 compared with controls (Fig. 2B; **P < 0.01). No significant differences in offspring heart mass were observed among females at PND3 and all groups at PND21 (Fig. 2C; *P* > 0.05).

Given that the reduced fetal heart mass in the hypothyroid group could be associated with activated programmed cell death and altered proliferation/differentiation factor expression, we evaluated *Bax* and *Bcl‑2* (apoptosis), proliferative activity (CDC47) and the transcription factors *Isl1*, *Fgf8* and *Foxh1*, which are implicated in cardiac development and cardiomyocyte lineage specification [23–29]. MH increased *Bax* expression versus control (Fig. 2G; ***P < 0.001). Maternal Kp10 administration restored pro‑apoptotic *Bax* expression to control levels (***P < 0.001), reducing the *Bax/Bcl‑2* ratio (*P < 0.05), and increased *Bcl-2* expression compared to control (*P < 0.05) (Fig. 2G-I). CDC47 immunolabeling showed lower cardiomyocyte proliferative activity in fetuses of MH rats versus controls (Fig. 2K-L; ****P < 0.0001), and maternal Kp10 administration restored it to control levels (*P* > 0.05). No differences were observed among groups for *Isl1*, *Fgf8* and *Foxh1* (Fig. 2J). However, no detectable expression of these transcripts was observed in the Kp10-treated group, in contrast to the hypothyroid group, in which expression was detected in half of the animals. Foxh1 expression was also not detected in animals from the control group (Fig. 2J).

### Maternal Hypothyroidism and Kp10 Administration Modulate Offspring Cardiac Expression of Redox Mediators and Endoplasmic Reticulum Stress Markers in a Sex- and Age-Dependent Manner

**Fig. 3:**
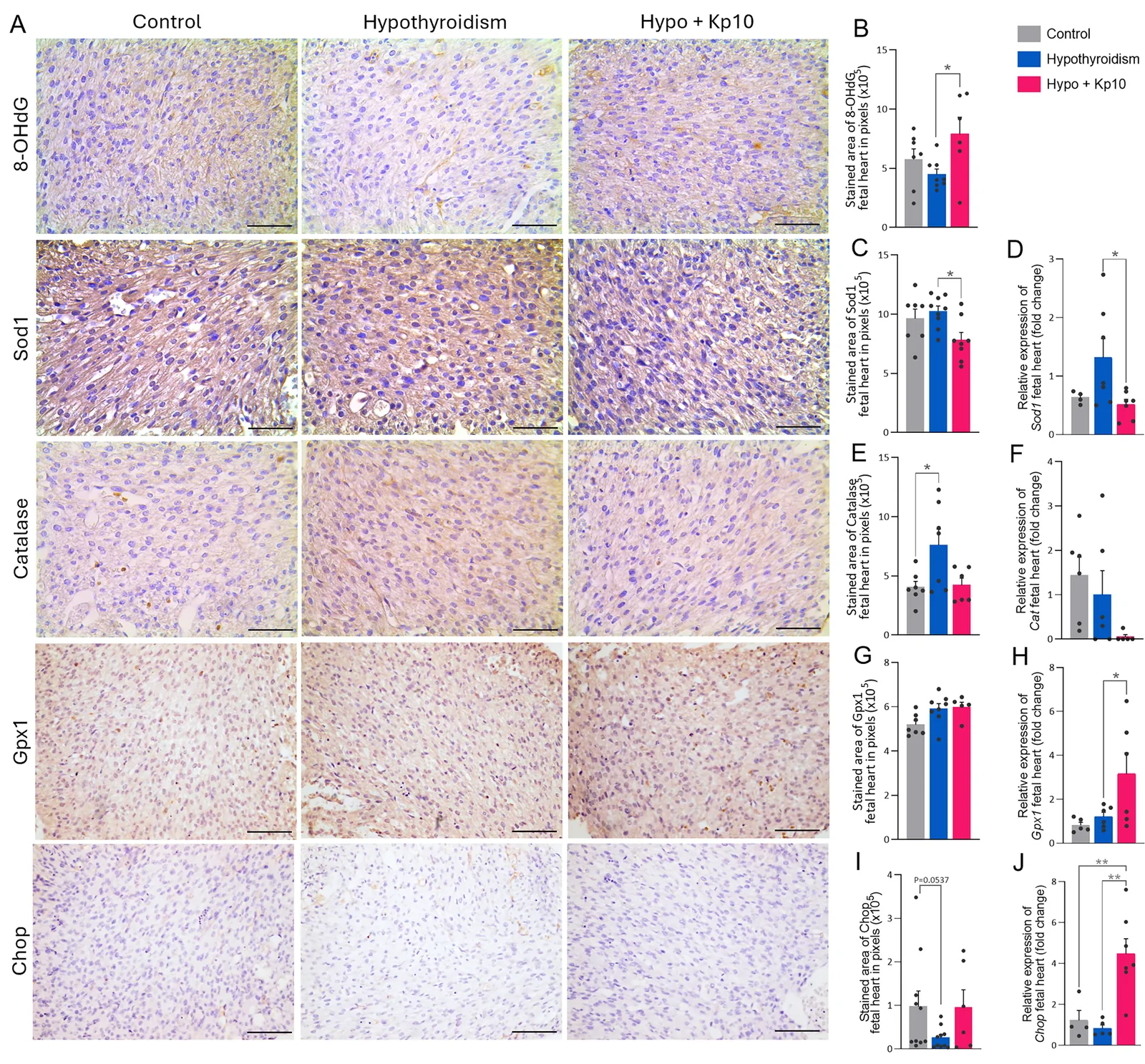
Expression of redox mediators in the hearts of offspring from the Control, Hypothyroidism and Hypothyroidism + Kp10 groups. **A** Photomicrographs of 8‑OHdG, Sod1, Catalase, Gpx1 and Chop immunohistochemical labeling in the GD18 fetal heart (streptavidin–biotin–peroxidase; Harris hematoxylin; Bar= 50 μm). **B** Immunolabeled pixel area of 8‑OHdG expression in the GD18 fetal heart (×10^5^) (*n* = 6–9/group). **C** Immunolabeled pixel area of Sod1 expression in the GD18 fetal heart (×10^5^) (*n* = 6–9/group). **D** Relative *Sod1* gene expression in the GD18 fetal heart (*n* = 4–7/group). **E** Immunolabeled pixel area of Catalase expression in the GD18 fetal heart (×10^5^) (*n* = 6–9/group). **F** Relative *Catalase* gene expression in the GD18 fetal heart (*n* = 5–6/group). **G** Immunolabeled pixel area of Gpx1 expression in the GD18 fetal heart (×10^5^) (*n* = 5–7/group). **H** Relative *Gpx1* gene expression in the GD18 fetal heart (*n* = 5–6/group). **I** Immunolabeled pixel area of Chop expression in the GD18 fetal heart (×10^5^) (*n* = 8–10/group). **J** Relative *Chop* gene expression in the GD18 fetal heart (*n* = 5–7/group) (Mean ± SEM). Significant differences are indicated by *P < 0.05, **P < 0.01, for one-way ANOVA followed by SNK post hoc test

Cardiovascular dysfunctions are often associated with cellular redox imbalance [30, 31], which may reflect altered fetal programming. To this end, we evaluated 8‑OHdG immunolabeling, a biomarker of oxidative DNA damage [32, 33], and the protein and gene expression of antioxidant enzymes (Sod1, Catalase and Gpx1) in the offspring heart. Regarding 8-OHdG expression, MH did not affect its levels in the fetal heart (Fig. 3A–B), whereas it reduced immunostaining in the hearts of male offspring at PND3 (Supplementary Fig. 1A–B; *P < 0.05). In contrast, maternal Kp10 administration increased 8-OHdG immunolabeling in the fetal heart compared with the hypothyroid group (Fig. 3B; *P < 0.05). However, at PND3 and PND21, no differences were observed compared with the other groups, regardless of sex (Supplementary Fig. 1E–F; *P* > 0.05).

**Fig. 4:**
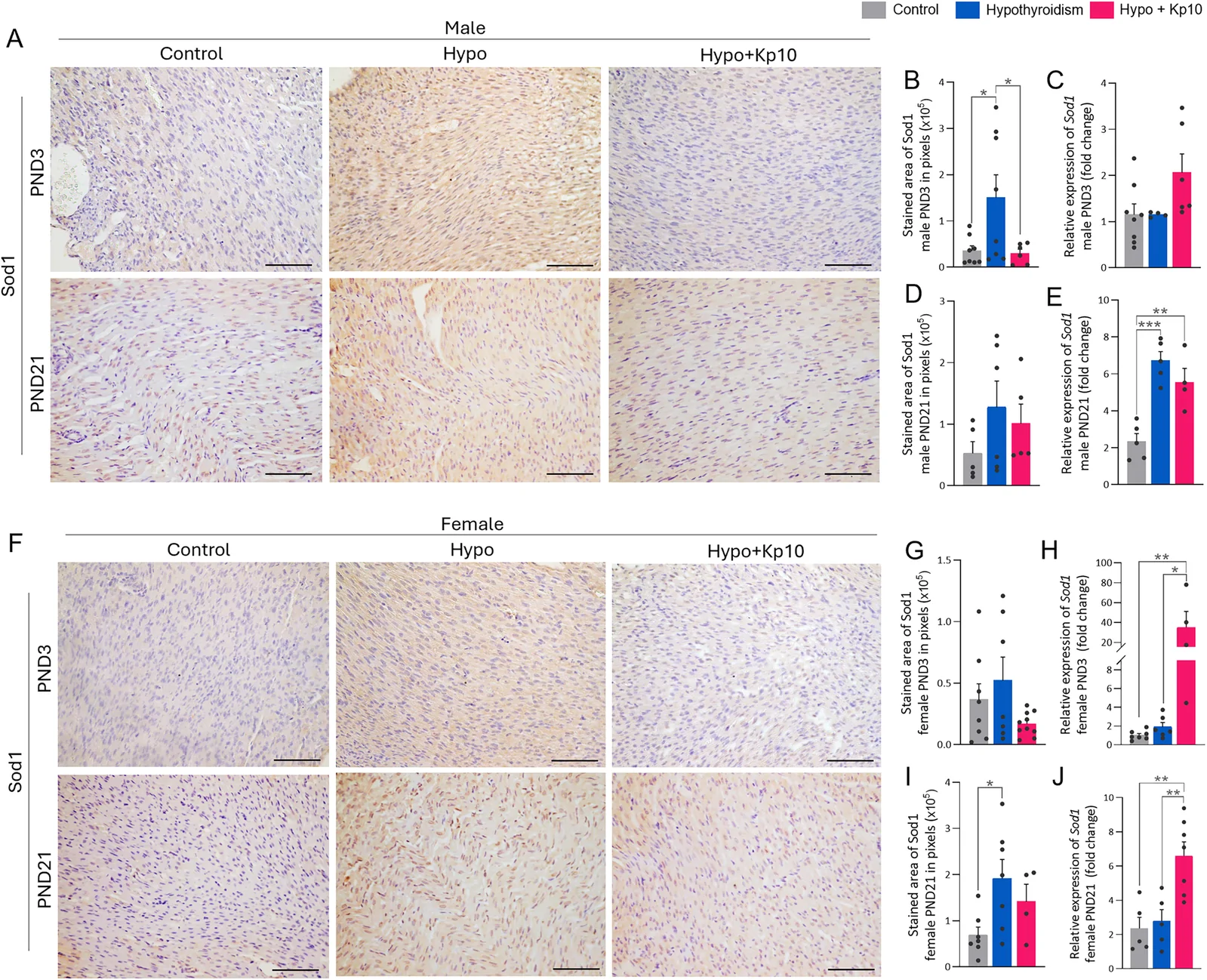
Expression of Sod1 in the hearts of offspring from the Control, Hypothyroidism and Hypothyroidism + Kp10 groups. **A** Photomicrographs of Sod1 immunohistochemical labeling in the male heart PND3 and PND21 (streptavidin–biotin–peroxidase; Harris hematoxylin; Bar = 50 μm). **B** Immunolabeled pixel area of Sod1 expression male heart PND3 (×10^5^) (*n* = 6–8/group). **C** Relative *Sod1* gene expression male heart PND3 (*n* = 3–6/group). **D** Immunolabeled pixel area of Sod1 expression male heart PND21 (×10^5^) (*n* = 5–6/group). **E** Relative *Sod1* gene expression male heart PND21 (*n* = 3–5/group). **F** Photomicrographs of Sod1 immunohistochemical labeling in the female heart PND3 and PND21 (streptavidin–biotin–peroxidase; Harris hematoxylin; Bar = 50 μm). **G** Immunolabeled pixel area of Sod1 expression female heart PND3 (×10^5^) (*n* = 6–10/group). **H** Relative *Sod1* gene expression male heart PND3 (*n* = 5–7/group). **I** Immunolabeled pixel area of Sod1 expression female heart PND21 (×10^5^) (*n* = 5–7/group). **J** Relative *Sod1* gene expression female heart PND21 (*n* = 5–7/group) (Mean ± SEM). Significant differences are indicated by *P < 0.05, **P < 0.01, and ***P < 0.001 for one-way ANOVA followed by SNK post hoc test

At GD18, Sod1 immunolabeling did not differ between control and hypothyroid groups; however, Kp10 administration reduced Sod1 expression compared with hypothyroid group (Fig. 3C; *P < 0.05), consistent with changes observed at the transcript level (Fig. 3D; *P < 0.05). After birth, MH increased the Sod1-labeled area in male and female hearts at PND3 and PND21, respectively, compared with controls (*P < 0.05), whereas Kp10 restored cardiac Sod1 expression in males at PND3 (Fig. 4A–B, F, I). MH also increased *Sod1* gene expression in male offspring at PND21 (***P < 0.001), while no effect of Kp10 administration was observed (Fig. 4E; **P < 0.01). In contrast, in female offspring, Kp10 increased *Sod1* mRNA expression compared with both control and MH groups at PND3 and PND21 (Fig. 4H, J; *P < 0.05, **P < 0.01).

For catalase, immunolabeling was higher in the hypothyroid group at GD18 compared with control group (Fig. 3E; *P < 0.05), whereas no difference was observed at the transcript level (Fig. 3F), despite the marked reduction observed in the Kp10-treated group. At PND3 and PND21, none or only weak catalase immunolabeling was detected in cardiomyocytes, regardless of the experimental group (Supplementary Fig. 2). Consistently, no *Cat* gene amplification was observed at PND3 or PND21.

**Fig. 5:**
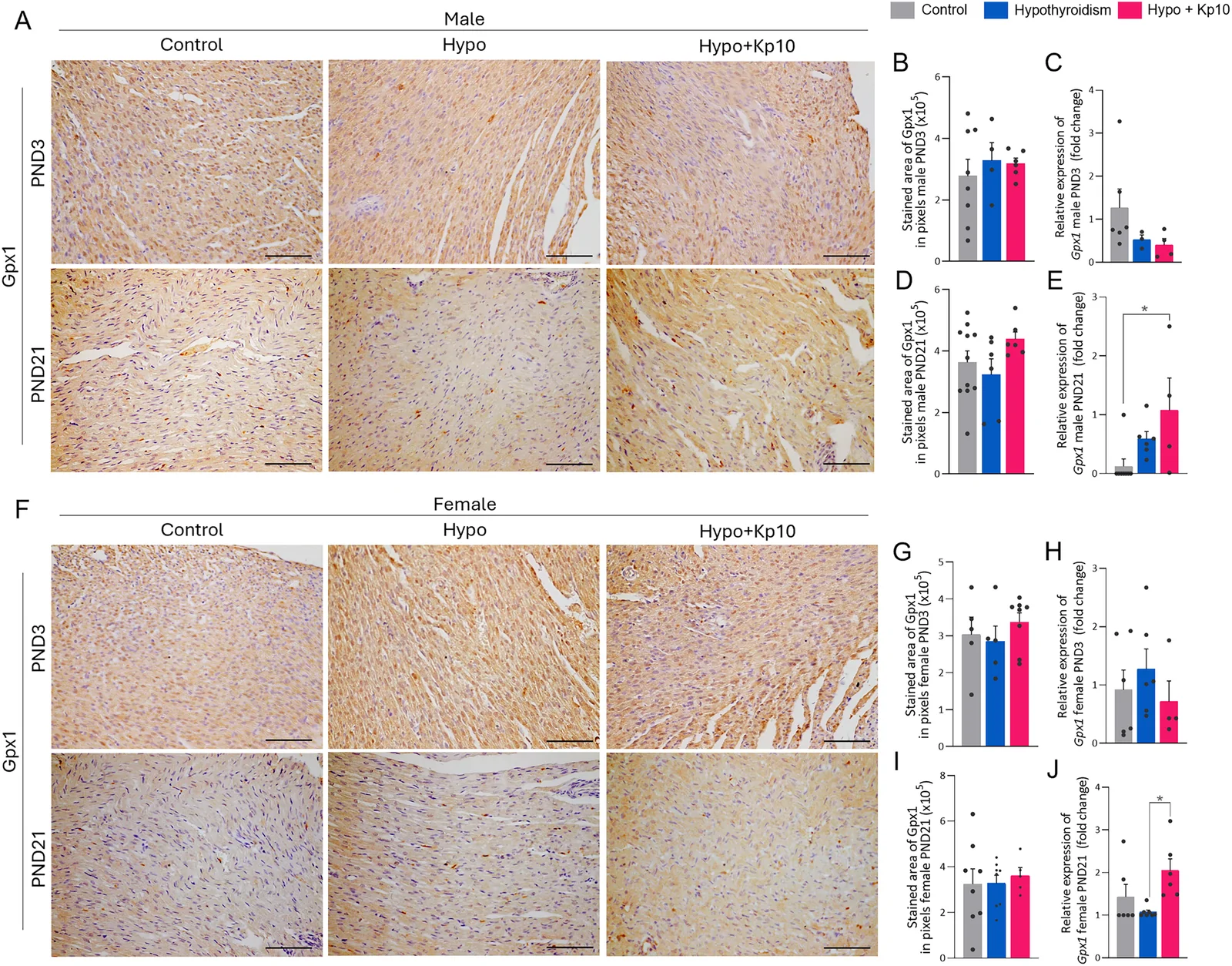
Expression of Gpx1 in the hearts of offspring from the Control, Hypothyroidism and Hypothyroidism + Kp10 groups. **A** Photomicrographs of Gpx1 immunohistochemical labeling in the male heart PND3 and PND21 (streptavidin–biotin–peroxidase; Harris hematoxylin; Bar = 50 μm). **B** Immunolabeled pixel area of Gpx1 expression male heart PND3 (×10^5^) (*n* = 5–8/group). **C** Relative *Gpx1* gene expression male heart PND3 (*n* = 3–6/group). **D** Immunolabeled pixel area of Gpx1 expression male heart PND21 (×10^5^) (*n* = 6–10/group). **E** Relative *Gpx1* gene expression male heart PND21 (*n* = 4–8/group). **F** Photomicrographs of Gpx1 immunohistochemical labeling in the female heart PND3 and PND21 (streptavidin–biotin–peroxidase; Harris hematoxylin; Bar = 50 μm). **G** Immunolabeled pixel area of Gpx1 expression female heart PND3 (×10^5^) (*n* = 5–8/group). **H** Relative *Gpx1* gene expression male heart PND3 (*n* = 6/group). **I** Immunolabeled pixel area of Gpx1 expression female heart PND21 (×10^5^) (*n* = 5–8/group). **J** Relative *Gpx1* gene expression female heart PND21 (*n* = 6/group) (Mean ± SEM). Significant differences are indicated by *P < 0.05 for one-way ANOVA followed by SNK post hoc test

Regarding Gpx1, no differences in immunolabeling were observed among groups in the fetal heart or at PND3 and PND21 (Figs. 3G and 5A–B, D, G and I). At the transcript level, however, Kp10 administration increased *Gpx1* expression at GD18 compared with the MH group (Fig. 3H; *P < 0.05). A similar increase was observed in male and female offspring at PND21 when compared with the control and hypothyroid groups, respectively (Fig. 5E, J; *P < 0.05).

**Fig. 6:**
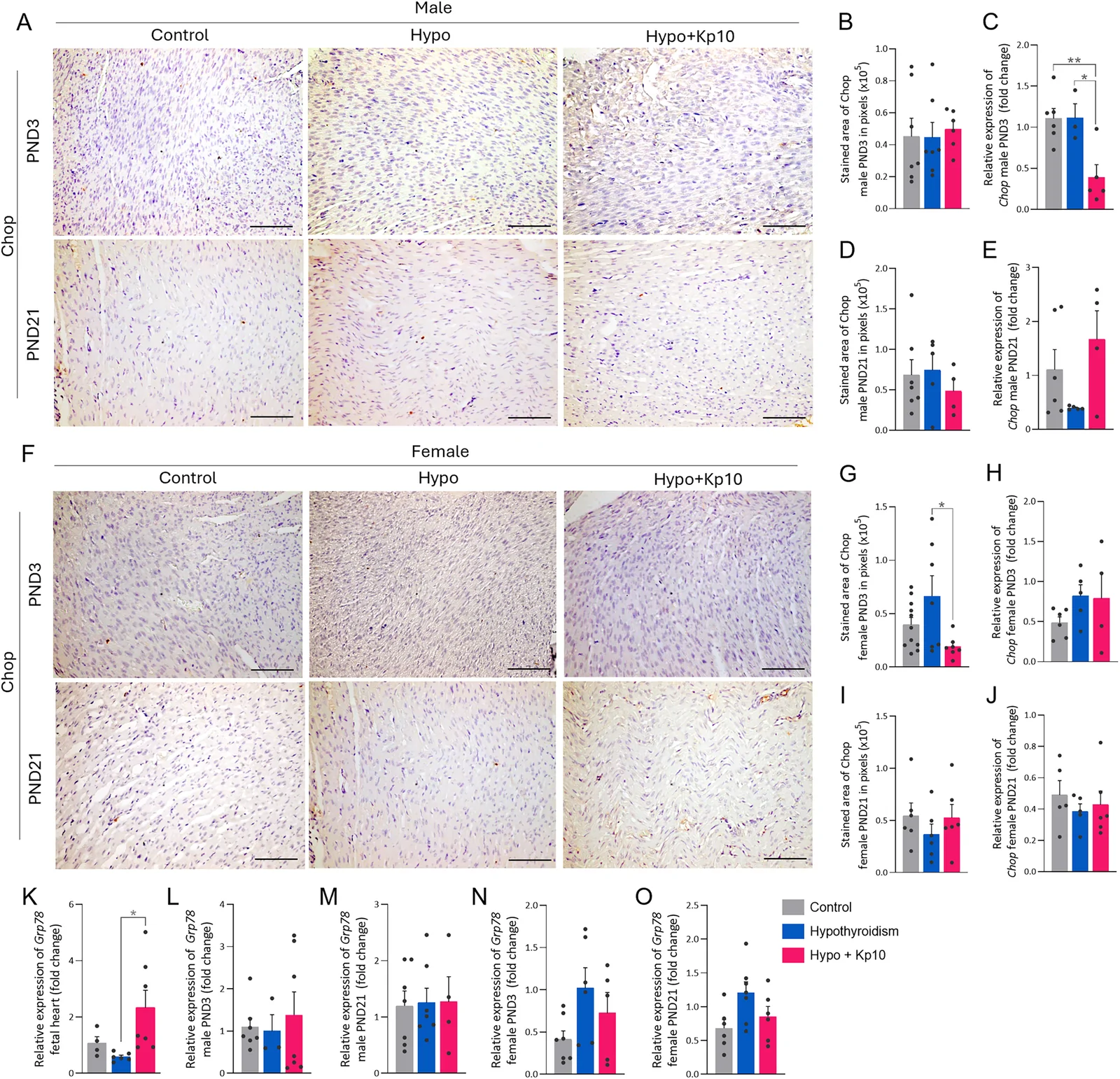
Expression of Chop in the hearts of offspring from the Control, Hypothyroidism and Hypothyroidism + Kp10 groups. **A** Photomicrographs of Chop immunohistochemical labeling in the male heart PND3 and PND21 (streptavidin–biotin–peroxidase; Harris hematoxylin; Bar = 50 μm). **B** Immunolabeled pixel area of Chop expression male heart PND3 (×10^5^) (*n* = 5–7/group). **C** Relative *Chop* gene expression male heart PND3 (*n* = 4–6/group). **D** Immunolabeled pixel area of Chop expression male heart PND21 (×10^5^) (*n* = 4–7/group). **E** Relative *Chop* gene expression male heart PND21 (*n* = 4–6/group). **F** Photomicrographs of Chop immunohistochemical labeling in the female heart PND3 and PND21 (streptavidin–biotin–peroxidase; Harris hematoxylin; Bar = 50 μm). **G** Immunolabeled pixel area of Chop expression female heart PND3 (×10^5^) (*n* = 7–11/group). **H** Relative *Chop* gene expression male heart PND3 (*n* = 4–6/group). **I** Immunolabeled pixel area of Chop expression female heart PND21 (×10^5^) (*n* = 5–6/group). **J** Relative *Chop* gene expression female heart PND21 (*n* = 5–6/group). **K** Relative *Grp78* gene expression in the GD18 fetal heart (*n* = 4–7/group). **L** Relative *Grp78* gene expression male heart PND3 (*n* = 3–6/group). **M** Relative *Grp78* gene expression male heart PND21 (*n* = 4–7/group). **N** Relative *Grp78* gene expression female heart PND3 (*n* = 5–7/group). **O** Relative *Grp78* gene expression female heart PND21 (*n* = 6/group) (Mean ± SEM). Significant differences are indicated by *P < 0.05 and **P < 0.01 for one-way ANOVA followed by SNK post hoc test

Considering that changes in cellular redox status can promote endoplasmic reticulum stress [32, 33], we also evaluated *Grp78* and Chop/*Chop* expression. At GD18, there was a trend toward reduced Chop immunolabeling in the hypothyroid group compared with controls (Fig. 3I; *P* = 0.0537), whereas maternal Kp10 administration increased the transcript levels of *Chop* (**P < 0.01) and *Grp78* (*P < 0.05) relative to control and/or hypothyroid groups (Figs. 3J and 6K). In contrast, at PND3, male offspring in the Kp10 group exhibited reduced *Chop* expression compared with both the hypothyroid (*P < 0.05) and control (**P < 0.01) groups (Fig. 6C), whereas female offspring in the Kp10 group showed reduced Chop immunolabeling compared with the hypothyroid group at the same postnatal age (Fig. 6G; *P < 0.05). No differences in *Grp78* expression were observed among groups at PND3 or PND21, nor in Chop/*Chop* expression at PND21 (Fig. 6D–E, I–J, L–O).

### Maternal Kp10 Administration Stimulates Fetal Cardiac Angiogenic Signaling and Restores Fetal and Postnatal Cardiac Ang2 Expression in a Sex‑ and Age‑Dependent Manner, which was Altered by Maternal Hypothyroidism

**Fig. 7:**
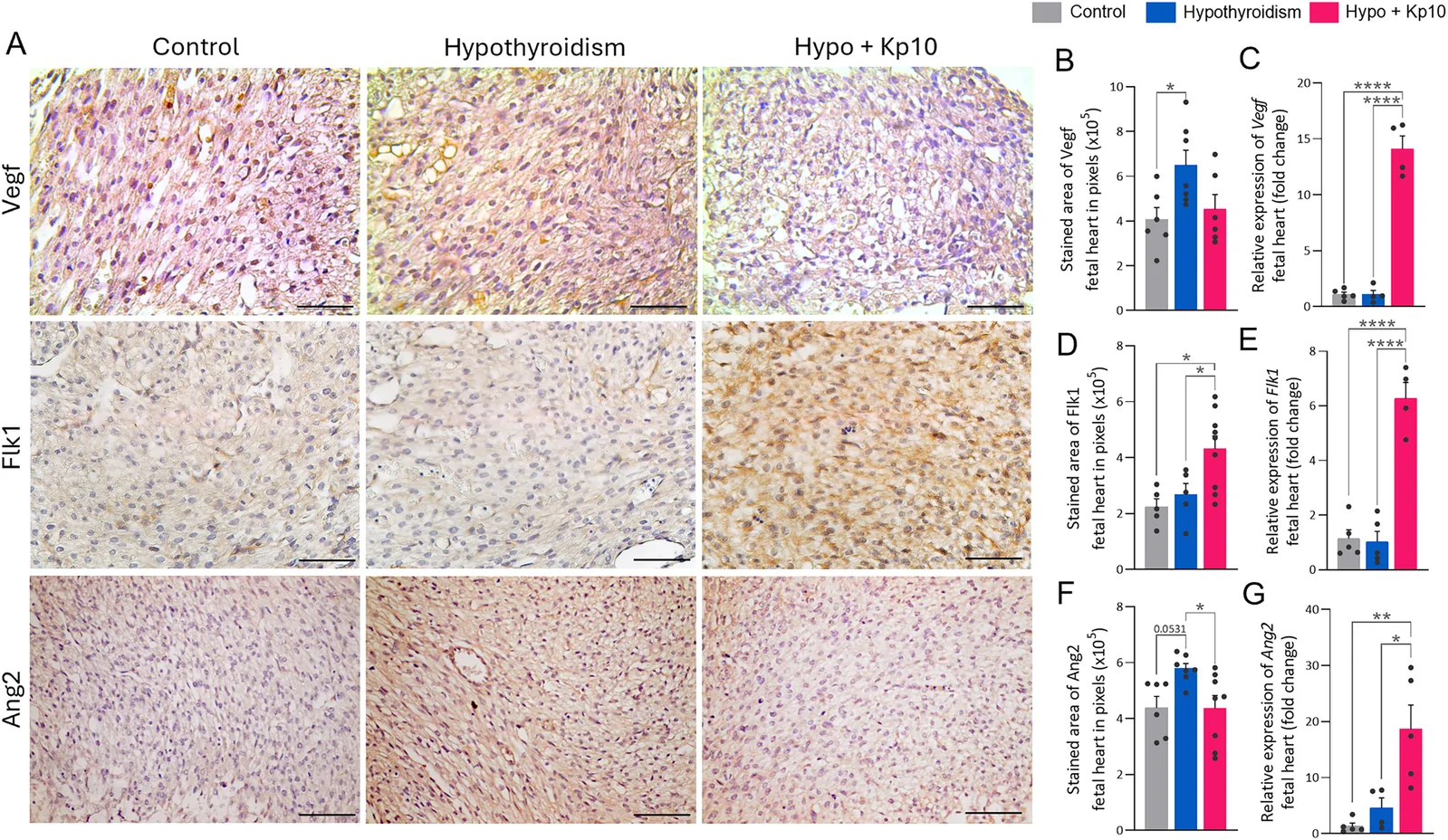
Expression of angiogenic factors (Vegf, Flk1 and Ang2) in the fetal hearts from the Control, Hypothyroidism and Hypothyroidism + Kp10 groups. **A** Photomicrographs of Vegf, Flk1 and Ang2 immunohistochemical labeling in the GD18 fetal heart (streptavidin–biotin–peroxidase; Harris hematoxylin; Bar = 50 μm). **B** Immunolabeled pixel area of Vegf expression in the GD18 fetal heart (×10^5^) (*n* = 6–9/group). **C** Relative *Vegf* gene expression in the GD18 fetal heart (*n* = 4–5/group). **D** Immunolabeled pixel area of Flk1 expression in the GD18 fetal heart (×10^5^) (*n* = 6–9/group). **E** Relative *Flk1* gene expression in the GD18 fetal heart (*n* = 4–5/group). **F** Immunolabeled pixel area of Ang2 expression in the GD18 fetal heart (×10^5^) (*n* = 6–9/group). **G** Relative *Ang2* gene expression in the GD18 fetal heart (*n* = 4–5/group) (Mean ± SEM). Significant differences are indicated by *P < 0.05, **P < 0.01, and ****P < 0.0001 for one-way ANOVA followed by SNK post hoc test

The development of cardiac microvasculature is often associated with intrauterine programming of cardiovascular diseases [34]. Therefore, gene and protein expression of Vegf, Flk1 (Vegfr2), and Ang2, essential for proper vascular development, were evaluated [35–37]. At GD18, MH increased cardiac Vegf immunolabeling (*P < 0.05) and showed a trend toward increased Ang2 expression (*P* = 0.0531) compared with controls (Fig. 7A–B, F). In contrast, maternal Kp10 administration restored MH-altered Ang2 levels (*P < 0.05) and attenuated Vegf immunolabeling (Fig. 7B, F). Additionally, offspring from the Kp10-treated group exhibited higher Flk1 expression than both control and hypothyroid groups at GD18 (Fig. 7D; *P < 0.05). At the transcript level, maternal Kp10 administration increased *Vegf* (****P < 0.0001), *Flk1* (****P < 0.0001), and *Ang2* (*P < 0.05, **P < 0.01) compared with control and hypothyroid groups (Fig. 7C, E, G).

**Fig. 8:**
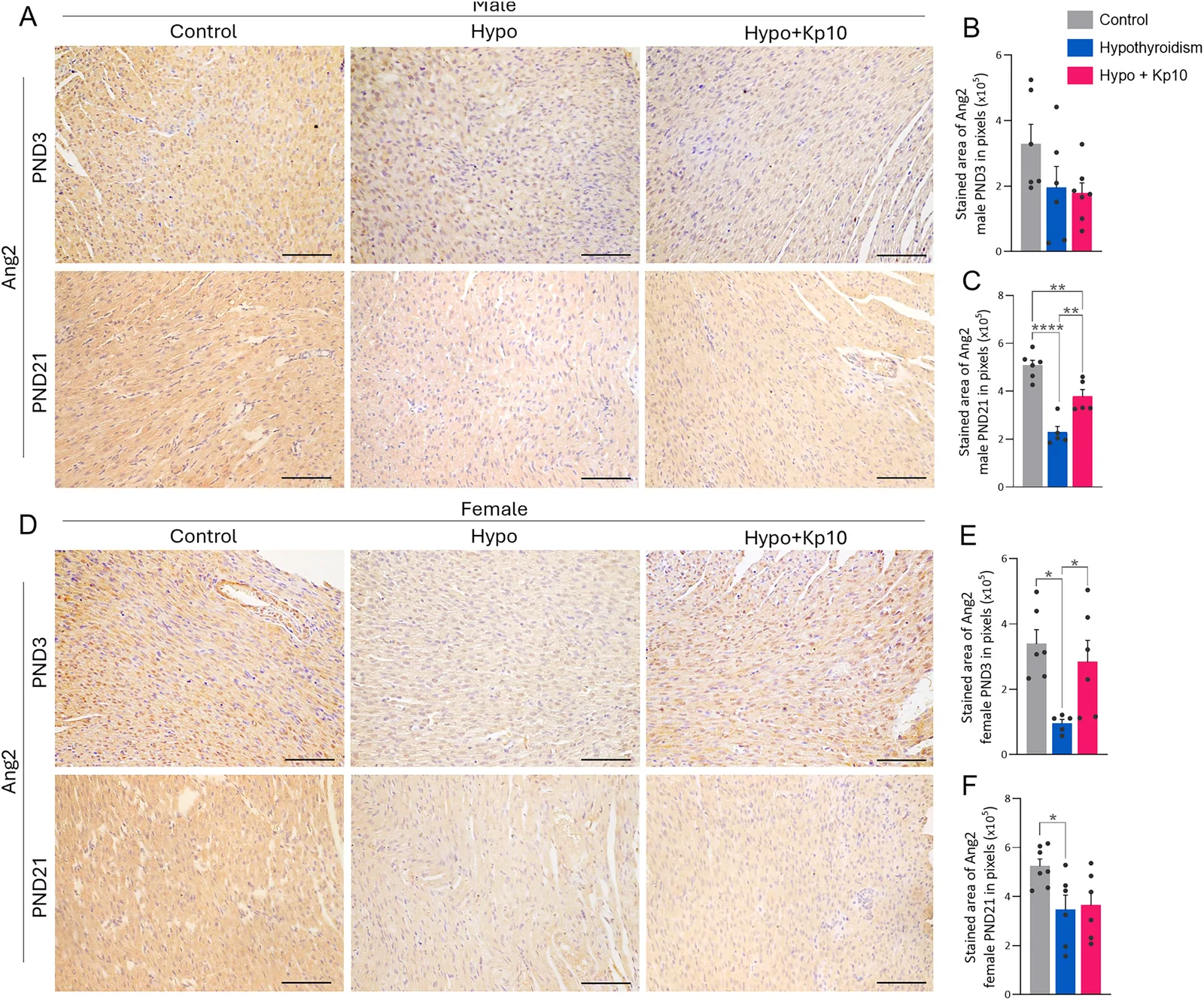
Expression of Ang2 in the hearts of offspring from the Control, Hypothyroidism and Hypothyroidism + Kp10 groups. **A** Photomicrographs of Ang2 immunohistochemical labeling in the male heart PND3 and PND21 (streptavidin–biotin–peroxidase; Harris hematoxylin; Bar = 50 μm). **B** Immunolabeled pixel area of Ang2 expression male heart PND3 (×10^5^) (*n* = 6/group). **C** Immunolabeled pixel area of Ang2 expression male heart PND21 (×10^5^) (*n* = 5/group). **D** Photomicrographs of Ang2 immunohistochemical labeling in the female heart PND3 and PND21 (streptavidin–biotin–peroxidase; Harris hematoxylin; Bar = 50 μm). **E** Immunolabeled pixel area of Ang2 expression female heart PND3 (×10^5^) (*n* = 5–6/group). **F** Immunolabeled pixel area of Ang2 expression female heart PND21 (×10^5^) (*n* = 5–7/group) (Mean ± SEM). Significant differences are indicated by *P < 0.05, **P < 0.01, and ****P < 0.0001 for one-way ANOVA followed by SNK post hoc test

After birth, immunohistochemistry revealed that Kp10 administration reduced Vegf immunolabeling in male offspring at PND21 compared with both the hypothyroid (**P < 0.01) and control (*P < 0.05) groups (Supplementary Fig. 3A, C), whereas no differences were observed among groups in female offspring (Supplementary Fig. 3D–F). Regarding Flk1, MH increased immunolabeling in males at PND3 compared with controls (Supplementary Fig. 4A–B; *P < 0.05), while no differences were observed among groups in female offspring (Supplementary Fig. 4D–F). Interestingly, MH markedly reduced Ang2 immunolabeling in females at PND3 compared with controls (Fig. 8D-E; *P < 0.05). At PND21, Ang2 expression was reduced in both sexes in the MH group relative to controls (Fig. 8A, C, F; *P < 0.05, ****P < 0.0001). Conversely, Kp10 administration increased Ang2 expression in female and male offspring compared with the hypothyroid group at PND3 (*P < 0.05) and PND21 (**P < 0.01), respectively (Fig. 8C, E).

### Hypothyroidism and Maternal Kp10 Administration did not Alter Cardiac Kiss1 Expression in the Offspring

To investigate whether the effects of maternal Kp10 administration on offspring cardiac development involved changes in the local Kiss1/Kiss1r system, we assessed *Kiss1/Kiss1r* gene and protein expression in fetal and postnatal hearts. qPCR analysis detected no *Kiss1r* amplification in cardiac tissues at any time point in any group, as well as negative Kiss1r immunostaining, although gene amplification and/or immunostaining were observed in rat placenta, liver, and adipose tissue used as positive controls. Immunohistochemical analysis revealed discrete Kiss1 expression restricted to the vascular endothelium, with no evident staining in cardiomyocytes or other cardiac compartments. This expression was observed only at postnatal stages (PND3 and PND21) (Supplementary Fig. 5A). Moreover, no differences in *Kiss1* transcript levels were observed among groups at GD18, PND3, or PND21 (Supplementary Fig. 5B–F).

## Discussion

This study demonstrated, for the first time, the deleterious effects of maternal hypothyroidism (MH) on cardiac developmental programming in rats, as well as the potential of maternal Kp10 administration to partially prevent these alterations. MH negatively affected fetal cardiac development by reducing cardiac mass, nuclear density, and cardiomyocyte proliferation, increasing pro-apoptotic factors, and dysregulating angiogenic mediators in the pre- and postnatal heart, with variations influenced by sex and developmental stage. Maternal Kp10 administration partially attenuated these alterations, restoring fetal cardiac proliferation and regulating pre- and postnatal cardiac Ang2 expression, suggesting novel mechanisms through which kisspeptin modulates and protects intrauterine cardiac programming.

Induction of MH compromised the offspring’s thyroid axis, as shown by reduced free T4 at PND3 regardless of sex. Although Kp10 had no statistically significant effect on maternal free T4 levels, our data suggests that administering Kp10 to hypothyroid dams improved offspring thyroid activity, as evidenced by free T4 levels at PND3 in the Kp10 group showing no difference compared to control. Although offspring of MH dams can exhibit congenital hypothyroidism [38, 39], this is the first study to suggest a protective role of Kp10 against congenital thyroid hypofunction, corroborating our previous hypothesis that kisspeptin modulates the thyroid axis in cyclic and pregnant hypothyroid rats [13, 40]. As expected, MH impaired fetal development, and hypothyroid offspring remained smaller until PND9. However, maternal Kp10 enhanced postnatal body mass gain, a pattern that persisted in females up to PND21. Although the protective effect of kisspeptin against IUGR‑related fetal growth alterations has not yet been elucidated, studies have shown that plasma kisspeptin levels are associated with positive pregnancy outcomes [13, 18, 41–43].

Gestational metabolic diseases, especially those leading to IUGR, are associated with increased cardiovascular risk throughout life [44]. In this study, MH reduced fetal heart mass, accompanied by decreased nuclear density and cardiomyocyte proliferation. Interestingly, although maternal Kp10 did not reverse reduced heart mass, it restored cardiomyocyte nuclear density and ameliorate the proliferative index in the fetal heart. Kisspeptin has been described as a modulator of cell proliferation in various tissues, mainly via direct administration in vascular and gonadal cells expressing Kiss1R [45–47]. However, this is the first study to demonstrate the positive modulatory effect of maternal kisspeptin administration on fetal organogenesis in a gestational disease.

Due to the intrinsic characteristics of cardiac tissue, fetal cardiomyocyte proliferation reflects the number of cardiomyocytes in the adult heart [22]. Moreover, dysregulated cell proliferation is associated with the onset of various heart diseases [48], although the underlying mechanisms remain incompletely elucidated. Besides reducing cell proliferation, MH increased *Bax* gene expression, a well‑known pro‑apoptotic factor [49] which may also explain the reduced fetal heart mass. In response, Kp10 administration reversed the high *Bax* expression, lowering the *Bax*/*Bcl2* ratio in fetal cardiac tissue, suggesting a role for kisspeptin in regulating proliferation and apoptosis during fetal development. Therefore, we investigated whether MH‑induced alterations could affect cell differentiation and maturation of fetal cardiac tissue, predisposing to cardiovascular disease later in life.

Although no significant differences were observed in transcripts promoting cardiomyocyte differentiation, offspring of hypothyroid dams treated with Kp10 did not express *Isl1*, *Fgf8*, or *Foxh1* in the heart, in contrast to fetuses from the hypothyroid group, which expressed all these genes. These factors are transcribed in finely orchestrated cascades to drive cardiomyocyte proliferation and differentiation [24–26]. Thus, considering *Foxh1*’s late expression and the reduced number of cardiomyocytes in MH fetuses, we suggest that MH temporally dysregulates differentiation waves in fetal cardiac tissue. Maternal Kp10 administration restored cardiomyocyte proliferation and appeared to stimulate differentiation, as fetuses in this group no longer expressed *Isl1*, *Fgf8*, or *Foxh1*, factors typically expressed during early cardiac development. However, further studies are required to confirm this hypothesis.

Most cardiovascular diseases involve an imbalance in cellular redox status [31], yet evidence within the context of fetal cardiac programming remains limited [30]. Here, we showed that 8-OHdG, a marker of oxidative DNA damage [21, 30, 31], was increased in fetal hearts of Kp10-treated animals, concomitant with reduced Sod1 gene and protein expression. Kp10 administration also increased Gpx1 gene expression in the fetal heart, suggesting a modulatory effect on fetal cardiac redox homeostasis. The increase in 8-OHdG following Kp10 administration may be explained by the recovery of the cardiomyocyte proliferative index, since cell proliferation increases local energy demand [50].

Interestingly, postnatally, Kp10-mediated modulation of redox status was sex- and age-dependent: it reduced MH-induced increases in Sod1 immunolabeling at PND3 in male offspring, while increasing *Sod1* gene expression at PND3 and PND21 in female offspring. In addition, in both sexes, Kp10 increased *Gpx1* mRNA expression at PND21. In contrast, MH modulated only Sod1 expression after birth, as, in addition to the increased Sod1 expression in males at PND3, both sexes exhibited increased gene or protein expression at PND21. Taken together, these data suggest that MH disrupts postnatal cardiac redox status, particularly by altering Sod1 expression, whereas maternal Kp10 administration modulates postnatal cardiac antioxidant enzyme expression predominantly in females.

Changes in cellular redox status can also activate the unfolded protein response (UPR), which initially aims to restore proper protein folding via Grp78 expression. If unresolved or excessive, ER stress ensues with Chop expression, potentially leading to cell death [51]. Accordingly, Kp10 increased *Grp78* and *Chop* gene expression in the fetal heart, possibly related to the observed rise in 8‑OhdG. However, after birth, Kp10 reduced Chop immunolabeling and gene expression in female and male offspring hearts, respectively, at PND3. These results suggest that maternal Kp10 administration activates the UPR in offspring hearts but exerts protective effects on this pathway in the neonatal heart. Indeed, several studies have shown kisspeptin’s modulatory role in ER stress activation [14, 52–57], including at the maternal–fetal interface in hypothyroid rats [14, 18, 19].

IUGR also compromises fetal angiogenesis by dysregulating vascular growth and proliferation factors [58, 59], thereby contributing to an adverse cardiovascular phenotype in adulthood [34]. In this study, we showed that MH alters angiogenic factor expression in the offspring heart. Specifically, Vegf and Ang2 immunolabeling were increased in fetal hearts of hypothyroid rats, whereas Ang2 immunolabeling was reduced in female offspring at PND3 and PND21 and in males at PND21. Conversely, maternal Kp10 administration reversed these alterations, restoring fetal and postnatal Ang2 protein immunolabeling, in addition to stimulating fetal cardiac *Vegf*, *Flk1*, and *Ang2* gene expression.

During heart development, angiopoietin-2 activity is required to induce angiogenesis [35], and its levels are regulated through complex interactions with Vegf and Tie2 [36, 37]. Notably, Ang2 has recently been proposed as a prognostic and therapeutic target for valvular heart disease, with significantly decreased circulating levels reported in affected patients [60]. Thus, our findings suggest that MH compromises angiogenesis in the fetal and postnatal heart by dysregulating Vegf, Flk1, and Ang2, whereas kisspeptin exerts potential cardioprotective effects on cardiac vascular development by positively regulating Ang2 expression.

As mentioned, kisspeptin acts directly via the KISS1R receptor [61]. However, in this study no Kiss1r gene and protein expression was detected in fetal or postnatal hearts. Although *Kiss1/Kiss1r* expression has been described in adult human and mouse hearts in one study [16], other reports have not detected *Kiss1r* expression in murine cardiac tissue [62–64]. Thus, our findings confirm this lack of expression and extend the literature by providing the first evaluation of *Kiss1/Kiss1r* expression in cardiac tissue during fetal development and lactation. It is important to note that MH‑induced placental dysfunction is associated with altered vascularization and angiogenic factor expression in the placenta [9, 14], which may alter fetal heart development and be linked to the vascular abnormalities seen in IUGR [65–68]. In this context, a recent study [69] using conditional *knockout* mice in trophoblast cells showed that defects in placental vascularization, particularly in the syncytiotrophoblast, promote congenital heart defects and increase embryonic lethality. Based on these data, our results and previous publications from our group [13, 14, 18, 19, 70] suggest that kisspeptin’s modulatory and protective effects on cardiac development occur, at least in part, via the placenta–heart axis, highlighting the influence of maternal health during gestation on cardiometabolic disease programming [71].

Our findings demonstrate that MH compromises fetal cardiac development by reducing cardiac mass, impairing cardiomyocyte morphology and proliferation, and dysregulating angiogenic factors in fetal and postnatal hearts in a sex- and age-dependent manner. Conversely, maternal Kp10 administration during gestation attenuated these alterations, restoring fetal cardiac proliferation and pre- and postnatal Ang2 expression, thereby suggesting novel protective mechanisms through which kisspeptin modulates intrauterine cardiac programming.

## Supplementary Information

Below is the link to the electronic supplementary material.

Supplementary Material 1

## Data Availability

No datasets were generated or analysed during the current study.
