# Supplementary material for: Maternal Kisspeptin-10 Treatment Partially Rescues Fetal and Postnatal Cardiac Programming Disrupted by Maternal Hypothyroidism
Source: Cardiovasc Toxicol. 2026 Sep 11;26(10):104. doi: 10.1007/s12012-026-10185-w (PMC13569559; doi:10.1007/s12012-026-10185-w)
Supplement: Supplementary file 1 [file 12012_2026_10185_MOESM1_ESM.docx]

**SUPPLEMENTARY INFORMATION**

**Fig S1.** Expression of 8-OHdG in the hearts of offspring from the Control, Hypothyroidism and Hypothyroidism + Kp10 groups. A) Photomicrographs of 8‑OHdG immunohistochemical labeling in the male heart PND3 and PND21 (streptavidin–biotin–peroxidase; Harris hematoxylin; Bar= 50µm). B) Immunolabeled pixel area of 8‑OHdG expression male heart PND3 (×10^5^) (n=6-9/group) (mean ± SEM). C) Immunolabeled pixel area of 8‑OHdG expression male heart PND21 (×10^5^) (n=6-9/group) (mean ± SEM). D) Photomicrographs of 8‑OHdG immunohistochemical labeling in the female heart PND3 and PND21 (streptavidin–biotin–peroxidase; Harris hematoxylin; Bar= 50µm). E) Immunolabeled pixel area of 8‑OHdG expression female heart PND3 (×10^5^) (n=6-9/group) (mean ± SEM). F) Immunolabeled pixel area of 8‑OHdG expression female heart PND21 (×10^5^) (n=6-9/group) (mean ± SEM). Significant differences are indicated by *P < 0.05 for one-way ANOVA followed by SNK post hoc test.

**Fig S2.** Expression of Catalase in the hearts of offspring from the Control, Hypothyroidism and Hypothyroidism + Kp10 groups. A) Photomicrographs of Catalase immunohistochemical labeling in the male heart PND3 and PND21 (streptavidin–biotin–peroxidase; Harris hematoxylin; Bar= 50µm). B) Photomicrographs of Catalase immunohistochemical labeling in the female heart PND3 and PND21 (streptavidin–biotin–peroxidase; Harris hematoxylin; Bar= 50µm). None or only weak catalase immunolabeling was detected.

**Fig S3.** Expression of Vegf in the hearts of offspring from the Control, Hypothyroidism and Hypothyroidism + Kp10 groups. A) Photomicrographs of Vegf immunohistochemical labeling in the male heart PND3 and PND21 (streptavidin–biotin–peroxidase; Harris hematoxylin; Bar= 50µm). B) Immunolabeled pixel area of Vegf expression male heart PND3 (×10^5^) (n=5-7/group) (mean ± SEM). C) Immunolabeled pixel area of Vegf expression male heart PND21 (×10^5^) (n=4-6/group) (mean ± SEM). D) Photomicrographs of Vegf immunohistochemical labeling in the female heart PND3 and PND21 (streptavidin–biotin–peroxidase; Harris hematoxylin; Bar= 50µm). E) Immunolabeled pixel area of Vegf expression female heart PND3 (×10^5^) (n=4-7/group) (mean ± SEM). F) Immunolabeled pixel area of Vegf expression female heart PND21 (×10^5^) (n=5-7/group) (mean ± SEM). Significant differences are indicated by *P < 0.05, **P < 0.01 for one-way ANOVA followed by SNK post hoc test.

**Fig S4.** Expression of Flk1 in the hearts of offspring from the Control, Hypothyroidism and Hypothyroidism + Kp10 groups. A) Photomicrographs of Flk1 immunohistochemical labeling in the male heart PND3 and PND21 (streptavidin–biotin–peroxidase; Harris hematoxylin; Bar= 50µm). B) Immunolabeled pixel area of Flk1 expression male heart PND3 (×10^5^) (n=5-7/group) (mean ± SEM). C) Immunolabeled pixel area of Flk1 expression male heart PND21 (×10^5^) (n=5-7/group) (mean ± SEM). D) Photomicrographs of Flk1 immunohistochemical labeling in the female heart PND3 and PND21 (streptavidin–biotin–peroxidase; Harris hematoxylin; Bar= 50µm). E) Immunolabeled pixel area of Flk1 expression female heart PND3 (×10^5^) (n=7-8/group) (mean ± SEM). F) Immunolabeled pixel area of Flk1 expression female heart PND21 (×10^5^) (n=5-6/group) (mean ± SEM). Significant differences are indicated by *P < 0.05, **P < 0.01, ****P < 0.0001 for one-way ANOVA followed by SNK post hoc test.

**Fig S5.** Expression of Kiss1 in the hearts of offspring from the Control, Hypothyroidism and Hypothyroidism + Kp10 groups. A) Photomicrographs of Kiss1 immunohistochemical labeling in the fetal heart GD18 (streptavidin–biotin–peroxidase; Harris hematoxylin; Bar= 50µm). B) Relative *Kiss1* gene expression fetal heart GD18 (n=7/group) (mean ± SEM). C) Relative *Kiss1* gene expression male PND3 (n=2-5/group) (mean ± SEM). D) Relative *Kiss1* gene expression male PND21 (n=3-4/group) (mean ± SEM). E) Relative *Kiss1* gene expression female PND3 (n=2-6/group) (mean ± SEM). F) Relative *Kiss1* gene expression female PND21 (n=3-5/group) (mean ± SEM).

**Fig S6.** Photomicrographs of the negative and positive controls for Sod1, Catalase, Gpx1, 8‑OHdG and Chop immunohistochemistry. A) Photomicrographs of immunohistochemistry for antioxidant enzymes and their respective positive controls: Sod1—placenta GD14; Catalase—decidua GD14; Gpx1—decidua GD14. B) Photomicrographs of immunohistochemistry for biomarkers of oxidative DNA damage and endoplasmic reticulum stress and their respective positive controls: Chop—placenta GD18; 8‑OHdG—placenta GD18. 40× objective.

**Fig S7.** Photomicrographs of the negative and positive controls for Vegf, Flk1, Ang2, p‑mTOR and Glut1 immunohistochemistry. A) Photomicrographs of immunohistochemistry for angiogenic factors and their respective positive controls: Vegf—placenta GD14; Flk1—placenta GD14; Ang2—fish ovary. 40× objective.

**Table S1.** Primary antibodies and incubation times for immunohistochemistry in hearts at GD18, PND3, and PND21.

| Antibody | Species/Clone | | Dilution | Incubation | Development time | Reference |
| --- | --- | --- | --- | --- | --- | --- |
| Cell proliferation and Kisspeptin system | | | | | | |
| anti-CDC47 | | Mouse/Monoclonal | 1:800 | 18 hours | 1 minute | sc-9966 |
| anti-Kiss1 | | Mouse/Monoclonal | 1:100 | 18 hours | 30 minutes | sc-101246 |
| anti-Kiss1R | | Rabbit/Polyclonal | 1:100 | 18 hours | 30 minutes | HPA071913 |
| Redox state | | | | | | |
| anti-SOD1 | | Mouse/Monoclonal | 1:1000 | 18 hours | 10 seconds | sc-101523 |
| anti-GPx1 | | Mouse/Monoclonal | 1:1000 | 18 hours | 45 seconds | sc-133160 |
| anti-catalase | | Mouse/Monoclonal | 1:1000 | 18 hours | 30 seconds | sc-271803 |
| anti-8-OHdG | | Mouse/Monoclonal | 1:400 | 18 hours | 10 minutes | sc-393871 |
| anti-CHOP | | Mouse/Monoclonal | 1:200 | 18 hours | 4 minutes | sc-71136 |
| Angiogenesis | | | | | | |
| anti-Vegf | | Rabbit/Polyclonal | 1:300 | 18 hours | 6 minutes | sc-152 |
| anti-Ang-2 | | Mouse/Monoclonal | 1:400 | 18 hours | 3 minutes | sc-74403 |
| anti-Flk1 | | Mouse/Monoclonal | 1:400 | 18 hours | 3 minutes | sc-6251 |

**Table S2.** Primer sequences used for mRNA analysis in hearts at GD18, PND3, and PND2.

| Gen | Sequence 3’-5’ | Accession number |
| --- | --- | --- |
| *Gapdh* | F: ACAGCCGCATCTTCGTTTGC  R: GCCTCACCCCATGTTATGTT | NM_017008.4 |
| *Kiss1* | F: GAGCCACTGGCAAAAATGGC  R: ATTAACGAGTTCCTGGGGTCC | NM_181692.1 |
| *Kiss1r* | F: CAACCTGCTGGCCCTATACC  R: TGCAGGGCGCCATCAGT | NM_023992.2 |
| *Isl1* | F: GATATGGGAGACATGGGCGA  R: GCACATTTCAAACATGCCGC | NM_017339.3 |
| *Fgf8* | F: CTCCAAGCCCAGCATGTGA  R: GAGCTGGTAGGTCCGGATGA | NM_133286.2 |
| *Foxh1* | F: GTAATTCAGGCCGCACCCTT  R: CCTTCCAGCCCTCATAGTCG | NM_001130493.1 |
| *Sod1* | F: GAAAGGACGGTGTGGCCAAT  R: CTCGTGGACCACCATAGTACG | NM_017050.1 |
| *Cat* | F: CTGACTGACGCGAGTTCCTA  R: GTGGTCAGGACATCGGGTTT | NM_012520.2 |
| *Gpx1* | F: GCGCTACAGCGGATTTGTTA  R: GAAGGCATACACGGTGGACT | NM_030826.4 |
| *Grp78* | F: TGAAGGGGAGCGTCTGAGTT  R: TCATTCCAAGTGCGTCCGAT | NM_013083.2 |
| *Chop* | F: TGGCACAGCTTGCTGAAGAG  R: TCAGGCGCTCGATTTCCT | NM_001109986.1 |
| *Vegf* | F: TCACCAAAGCCAGCACATAG  R: TTTCTCCGCTCTGAACAAGG | NC_000083.7 |
| *Ang2* | F: TCCAGACTGACGCACATCAC  R: ATTTCTCCAGACCCGCAGTG | NM_134454.1 |
| *Kdr (Flk1)* | F: GGTGGGATGATGCCCAGTAAA  R: CTGCAACATCCACCAGCTTT | NM_013062.2 |
|  |  |  |
